# The importance of an individual approach to Graves’ hyperthyroidism

**DOI:** 10.1186/1756-6614-8-S1-A2

**Published:** 2015-06-22

**Authors:** Tomasz Bednarczuk

**Affiliations:** 1 Department of Internal Medicine and Endocrinology, Medical University of Warsaw, Warsaw, Poland

## 

Graves' disease (GD) is an autoimmune disorder characterized by the presence of circulating autoantibodies that stimulate the thyroid hormone receptor (TSHR), resulting in hyperthyroidism and goiter. GD may affect also other organs, leading to Graves' orbitopathy, Graves' dermopathy and acropachy. It is likely that the extrathyroidal manifestations of GD are due to autoimmunity against antigens common to the thyroid and other affected organs (for example TSHR). Although its exact etiology remains to be established, GD is believed to result from a complex interaction between genetic, environmental and endogenous factors.

The clinical picture of GD is highly variable. Measurements serum levels of anti-TSHR autoantibodies (TRAb) and thyroid ultrasonography represent the most important diagnostic tests for GD.

Existing treatment modalities for Graves' hyperthyroidism includes antithyroid drugs (ATDs), radioactive iodine, and surgery. The use of ATDs as the initial treatment option in GD is well accepted. However, the optimal treatment duration and the predictive marker of remission after ATDs therapy are still controversial. A high relapse rate after a course of ATDs (60-70%), implies the use of ablative treatments (radioactive iodine or surgery) that remove or decrease thyroid tissue leading to lifelong hypothyroidism. There is a lack of general agreement as to which therapy for Graves’ hyperthyroidism is the best as none is ideal and all may have severe side effects. Moreover, none of these treatments targets the autoimmune disease process. Therefore the treatment plan should be established individually and carefully discussed with the patient. Hopefully novel agents that might act on the autoimmune disease process will be approved for Graves' hyperthyroidism.

